# Trueness and depth discrepancy of post-space scans using an intraoral scanner: influence of preparation dimensions: an in vitro study

**DOI:** 10.1186/s12903-025-06062-7

**Published:** 2025-05-10

**Authors:** Mostafa Shahin Zaki, Cherif Adel Mohsen, Mostafa Elhusseiny Mohamed

**Affiliations:** Fixed Prosthodontics Department, Faculty of Dentistry, Minia University, Misr Aswan Agricultural Rd. ،ARD SHALABY ،ELMinia, Minia, Egypt

**Keywords:** Dental impression technique, Post and core technique, Polyvinyl siloxane

## Abstract

**Background:**

Intraoral scanning of post spaces offers a potential alternative to conventional impressions, but its effectiveness depends on overcoming limitations influenced by post space dimensions.

**Aim of the study:**

This study aimed to evaluate the trueness and depth discrepancy percentage of scanned post spaces with different dimensions using an intraoral scanner.

**Materials and methods:**

Twenty single-rooted human maxillary canines were endodontically treated. Teeth were assigned to two post space preparation width groups: N (Ø1.5 mm) and W (Ø1.7 mm) (*n* = 10 per group). Each width group was further subdivided into two depth subgroups: S (6 mm) and L (10 mm) (*n* = 5 per subgroup). This resulted in four experimental subgroups: NS (Ø1.5 mm, 6 mm), NL (Ø1.5 mm, 10 mm), WS (Ø1.7 mm, 6 mm), and WL (Ø1.7 mm, 10 mm). Specimens were scanned using the Panda P2 intraoral scanner (IOS), and the obtained STL files were aligned and compared with those from traditional impressions scanned with the InEos X5. Trueness and depth discrepancy percentage were evaluated using reverse engineering software. The data were statistically analysed using a Two-Way ANOVA, followed by multiple pairwise comparisons using Tukey’s HSD for each individual factor.

**Results:**

Preparation width had no significant effect on trueness (*p* > 0.05), whereas increasing preparation depth significantly reduced it. Additionally, a greater preparation width significantly decreased the depth discrepancy percentage, while increasing preparation depth led to a significant increase. A statistically significant, very strong positive correlation was observed between RMS and depth discrepancy percentage (*r* = 0.898), indicating that greater deviations in trueness were associated with increased depth discrepancies.

**Conclusions:**

Within the study’s limitations, trueness improved by reducing post space depth and remained clinically acceptable for all subgroups. However, increasing depth to 10 mm raised the depth discrepancy beyond the clinically acceptable range, while decreasing width also increased discrepancy.

**Clinical trial number:**

Not applicable.

## Background

After root canal treatment, the risk of root fractures can be attributed to the reduction in water content, which causes dentin shrinkage stresses that initiate cracks, eventually leading to tooth fractures [1]. This risk is further compounded by the increased loss of tooth structure due to access gained for pulp and caries lesions, which may undermine cusps, ridges, and impair the structural integrity of the tooth. A post is required for tooth reconstruction and retention of fixed dental prostheses [2–7].

Root canals often exhibit varied morphologies that can impact the restoration process, including oval shapes, cavities, excessive preparation from previous restorations, over-instrumentation, incomplete root formation, internal resorption, and developmental anomalies [8]. The use of prefabricated posts requires root canal preparation to accommodate a standardized post, which may weaken the tooth structure. Additionally, the uneven gap between the post and dentin increases the risk of fracture and debonding [9].

Several approaches have been proposed to eliminate or reduce the discrepancy between root canal anatomy and prefabricated posts. These include filling the root canal with composite resin to accommodate the canal’s anatomy. Another direction involves using anatomically shaped posts as the gold standard in widely flared root canals, considering adhesion, where bubble and gap formation during cementation can be significantly reduced [10, 11].

Custom posts, made from resin composites or hybrid materials with an elastic modulus and shade similar to dentin, can serve as viable alternatives to prefabricated post systems, minimizing both mechanical and esthetic risks. When modifying a post system, preserving dental structure and avoiding excessive removal of root dentin are essential. Fabricating patient-specific posts ensures an optimal anatomical fit while minimizing unnecessary dentin removal [12–15].

Custom posts can be fabricated using either direct or indirect techniques. The direct method involves shaping an acrylic resin pattern directly within the root canal, while the indirect method relies on an elastomeric impression to create a stone cast of the canal. Both techniques have inherent limitations, including polymerization shrinkage of acrylic resin, dimensional instability of gypsum materials, technique sensitivity, potential residual resin debris within the canal, and the additional clinical and laboratory costs associated with fabrication [16–19].

Commercially available intraoral scanners now accommodate diverse clinical needs, with manufacturers continually refining their capabilities. Initially limited to scanning single crowns and inlay/onlay restorations, these scanners can now capture both soft and hard tissues for a wide range of restorations [20–25].

Despite limited research on the accuracy of different intraoral scanners (IOSs) in capturing post-space impressions, existing studies have yielded inconsistent findings. Hendi et al. [13] investigated the retention of posts and cores fabricated using digital and conventional impression techniques, concluding that conventional impressions provided superior retention. Similarly, Kanduti et al. [26] observed greater discrepancies in the apical region than in the cervical region when comparing digital and conventional impressions. Pinto et al. [27] examined impression quality at post-space depths of 8.8 mm and 9.5 mm using IOSs and conventional silicone impressions, reporting significant discrepancies in digital impressions. Elter et al. [28] further assessed the accuracy of various IOSs in recording post-space depths, noting a decline in accuracy as depth increased beyond 20 mm.

Few studies have specifically investigated depth discrepancy in intraoral scanner (IOS) impressions of post spaces, and those available have assessed limited depth ranges without systematically evaluating its effect on trueness. Most research has primarily compared digital and conventional impression techniques, with less focus on the factors influencing IOS trueness in post-space scanning. Additionally, the effect of post-space width on trueness remains largely unexplored, despite evidence suggesting that cervical diameter influences scanning accuracy [29]. Variations in IOS systems and study methodologies have also led to inconsistent findings, making it difficult to establish a clear consensus on the reliability of digital impressions for post-space recording.

Recent advancements in intraoral scanner (IOS) technology have significantly enhanced their precision, speed, and depth capture capabilities, establishing them as integral tools in digital dentistry [30–33]. The Panda P2 intraoral scanner is designed to capture deep preparations up to 15 mm, with an adjustable depth extension to 20 mm. The device demonstrates a trueness of less than 15 μm and a precision of up to 10 μm, ensuring high accuracy in digital impressions [34].

The primary objective of this in vitro study was to evaluate the trueness and depth discrepancy percentage for scanned post spaces of different dimensions using an intraoral scanner. The null hypothesis of this study stated that there would be no significant difference in trueness and depth discrepancy percentage, regardless of post space preparation width and depth.

## Materials and methods

This study received ethical approval from the Research Ethics Committee of the Faculty of Dentistry, Minia University, Egypt, under approval number Committee No. 95, Registration No. 736, Date: 28/03/2023. Twenty human maxillary canines extracted for periodontal reasons were obtained from the Oral Surgery Department, Faculty of Dentistry, Minia University. Inclusion criteria were based on root dimensions, predominantly straight roots with labio-palatal dimensions of 6 to 7 mm, mesio-distal dimensions of 5 to 6 mm, and anatomical length of 22 to 23 mm. Visual and radiographic assessments confirmed the absence of prior endodontic treatment, restorations, caries, cracks, or internal resorption. Only teeth with straight, single root canals and fully matured apices were included. Cracks were identified using a 6x magnifying loupe and LED trans-illumination, with light diffraction at crack sites ensuring accurate detection and exclusion of compromised specimens [35–40]. Teeth were cleaned of soft tissue attachments, immersed in sodium hypochlorite solution for 7 days, and then preserved in saline solution at room temperature to prevent desiccation [41].

A power analysis was conducted using G*Power software (version 3.1.9.7; Heinrich Heine University, Düsseldorf) [42] to determine the optimal sample size, with an alpha level of 0.05, a statistical power of 80%, and an effect size of 1.23 derived from an internal pilot study. The pilot study included twelve single-rooted canines, categorized by post space width (N: Ø1.5 mm, W: Ø1.7 mm, *n* = 6 per group) and depth (S: 6 mm, L: 10 mm, *n* = 3 per subgroup), with calculated means and standard deviations as follows: NS (60.66 ± 6.12), NL (84.61 ± 9.17), WS (45.37 ± 6.35), and WL (73.88 ± 6.80). The analysis indicated that a total of nine teeth distributed across the study subgroups were required to achieve 80% power with an alpha error of 0.05. However, to enhance statistical robustness and ensure balanced representation across all experimental conditions, the total sample size was increased to 20 teeth. A post-hoc power analysis confirmed that this adjustment maintained a statistical power exceeding 80%, reinforcing the reliability of the study.

### Specimens’ Preparation

The teeth were sectioned approximately 2 mm coronal to the cement-enamel junction using a double-sided diamond disk mounted on a low-speed handpiece, with continuous coolant flow to prevent overheating. Teeth were endodontically treated using E-Flex (Eighteeth, District, Changzhou City, China) 20# 0.06, 25# 0.06, 30# 0.04, and 35# 0.04 as the master apical files. Irrigation was performed between files using 5.25% sodium hypochlorite solution, 17% EDTA solution, followed by saline irrigation as a final solution. Canals were dried with paper points (35# 0.02), and Well-Root (Well-Root ST, Vericom, Gangwon-Do, Korea) bio-ceramic sealer was injected into the root canals. The master cone (Diadent Group International, Korea) 35# 0.04 was inserted, followed by cold lateral condensation of the obturation material for all teeth [43].

Teeth were mounted into acrylic resin resin (Acrostone Acrylic Material-Cold Cure; ACROSTONE Co) to a level 2 mm apical to the CEJ to simulate gingival color [44, 45]. 

### Grouping of the specimens

All 20 specimens were randomly allocated for post space preparations based on different cervical widths and depths. Randomization was performed using a statistical website (Randomizer.org). Each specimen was assigned a unique identifier, and a block randomization approach was used to maintain balance across the subgroups. The allocation sequence was concealed, and the assignments were only revealed at the time of post-space preparation to prevent allocation bias. The study included the following four subgroups:

- NS: Preparation width of 1.5 mm and depth of 6 mm.
- WS: Preparation width of 1.7 mm and depth of 6 mm.
- NL: Preparation width of 1.5 mm and depth of 10 mm.
- WL: Preparation width of 1.7 mm and depth of 10 mm.

### Post space Preparation

**Fig. 1:**
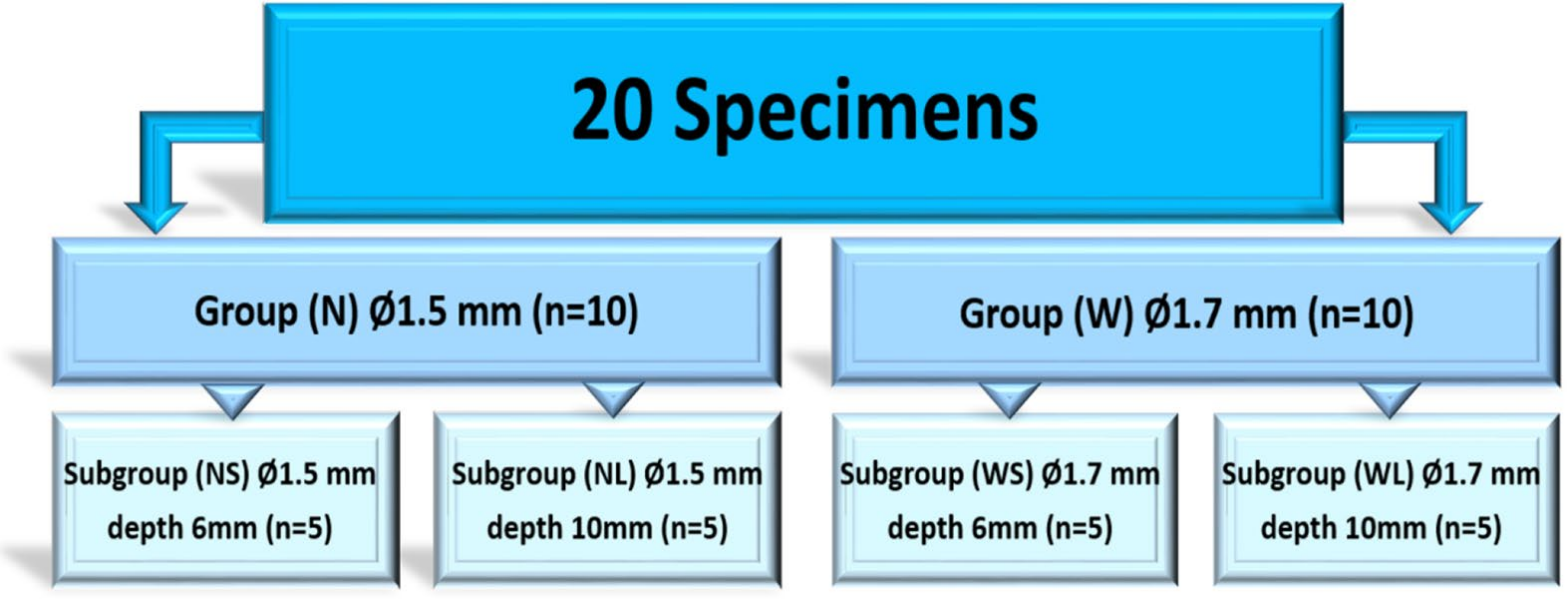
Flowchart of the study design

Water-cooled sequential drilling was performed using Peeso reamers (NORDIN, Switzerland). For the Ø1.5 mm preparation width (Group N), drilling progressed from Peeso reamer No. 1 to No. 5. For the Ø1.7 mm preparation width (Group W), enlargement continued with Peeso reamer No. 6. Preparation depth was set at 6 mm for subgroups NS and WS, while for subgroups NL and WL, the depth was set at 10 mm (Fig. 1).

### Scanning of the post space

The optical scanners used in this study are listed in Table 1. Samples were grouped and numbered within all subgroups to facilitate data entry. The Panda P2 intraoral scanner (Pengtum Technologies, Shanghai, China) was used to scan post spaces for all subgroups. A calibration process was performed and repeated before scanning each new subgroup, according to ISO 5725-1:2023. To ensure repeatability, calibration was performed before each subgroup scan to exclude any potential influence of calibration factors on scanning accuracy [46, 47].

**Table 1:** Optical scanners

Optical Scanner	Manufacturer	Scanning tip	Acquisition technology
InEos X5	Dentsply Sirona	Robotic Arm	Digital light stripe technology
Panda P2	Pengtum Technologies	-19.6 × 14.6 mm -35, 45 and 55-degree angles.	Continuous stereographic photography

**Fig. 2:**
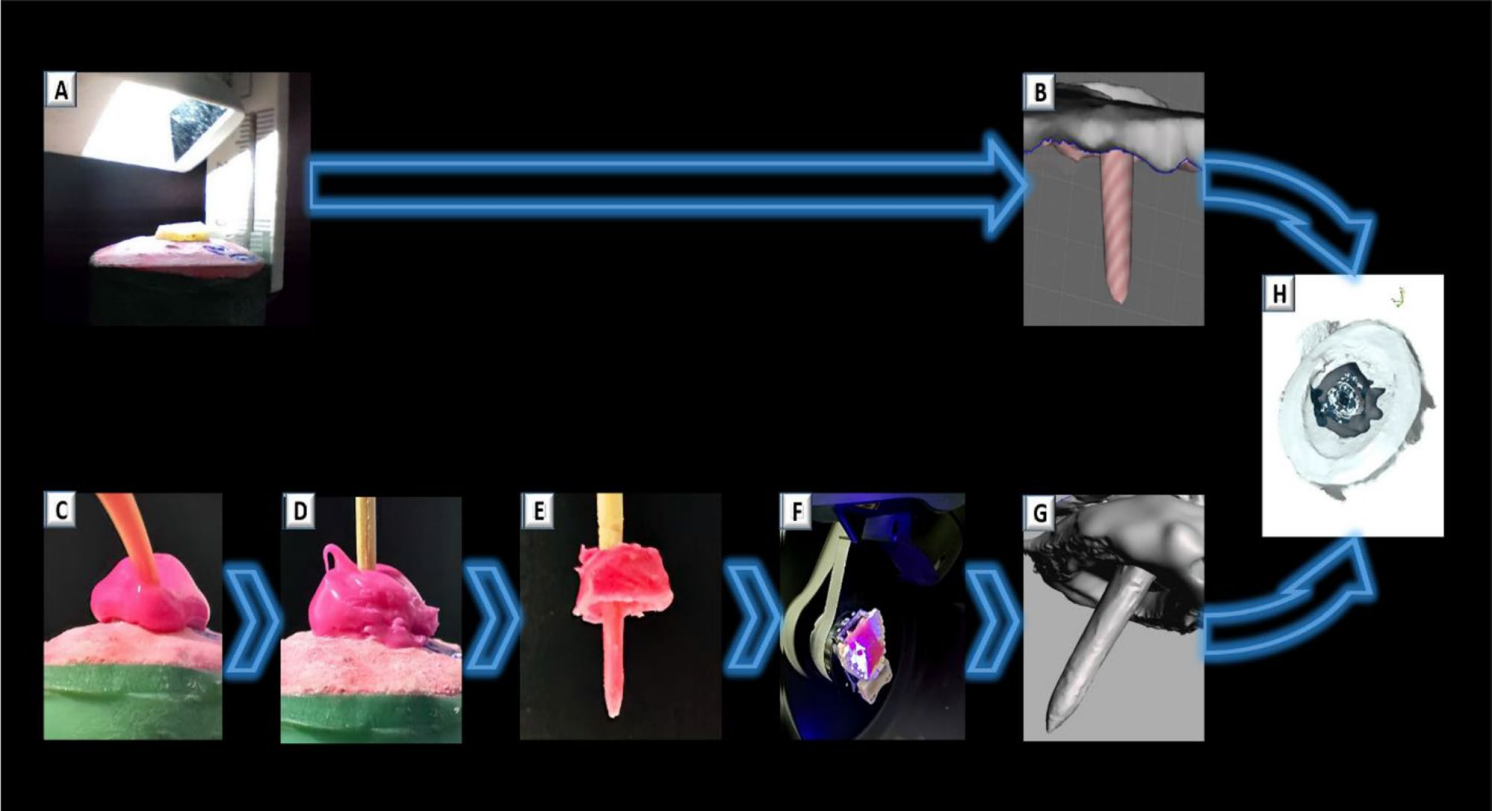
Flow diagram of the study. **A**. Post space scanning using Panda P2 IOS; **B**. Panda P2 IOS scan model; **C**. Injecting light-body polyvinyl siloxane impression material; **D** and **E**. Adjusted toothpick supporting post space impression; **F**. Reference scanning of post space impressions using InEos x5 desktop scanner; **G**. Reference scan model; **H**. Reference best fit alignment

All samples were fixed firmly to the desktop surface. At the acquisition page, the maximum scan depth (Deep Gear) was selected. At room temperature, an experienced operator performed the scanning to minimize operator bias. The scanner was held over the occlusal surface at a 10 mm distance. Starting from the occlusal notch, a clockwise motion was followed to capture the post space circumferential depth (Fig. 2A). After scanning and rendering the model, the data were exported in STL file format (Fig. 2B) [45, 48].

### Impression of post spaces

A conventional impression of the post spaces was made for all samples using light-body polyvinyl siloxane impression material (Hydrorise Light Body, Zhermack, Italy). A fine intraoral plastic tip, mounted on a plastic mixing tip, was used to inject the impression material into the root canal (Fig. 2C). Gentle air blow was applied to minimize void entrapment. A wooden toothpick, previously prepared to fit loosely according to the different preparation depths and widths, was inserted to support the impression material and minimize dimensional changes. The additional material was then injected to cover the prepared tooth (Fig. 2D, E) [28].

### Reference scanning of post space impression

Reference STL files were generated by scanning the impressions of all samples using the InEos X5 desktop scanner (Dentsply Sirona, Bensheim, Germany). The post space impression was securely attached to a plaster model using wax. The assembly was then fastened to the model holding plate, which was firmly secured by the robotic arm of the device (Fig. 2F). Scanning was performed in high dynamic range (HDR) mode, with the maximum calculation time set to the highest value by selecting the “complete reconstruction model” option. Once scanning was completed and the model was rendered, the data were exported in STL file format (Fig. 2G) [28, 45].

### Trueness measurement

Trueness measurements were obtained using the reverse engineering software Geomagic Control X 2024 (Geomagic, 3D Systems Manufacturing, Rock Hill, USA). Each reference STL file, obtained from the InEos X5 desktop scanner, was superimposed onto its corresponding STL file from the intraoral scanner. Dataset alignment began with an initial alignment step, followed by the reference best fit alignment algorithm (Fig. 2H). A 3D comparison was then performed by selecting the area of interest and applying a 100% sampling ratio, the shortest projection direction, and automatic estimation of maximum deviations. The comparison settings were confirmed before proceeding. After alignment, the square of the 3D phase difference between corresponding points was calculated. The root mean square (RMS) was calculated as the square root of a value obtained by dividing the sum of squares by the number of points, applying the following equation:-

$$\:\text{R}\text{M}\text{S}=\:\frac{1}{\sqrt{n}}\:\times\:\:\sqrt{\sum\:_{i=1}^{n}{\left({x}_{1i}-{x}_{2i}\right)}^{2}}$$

x_1i_: measurement of point i on the reference scan.

x_2i_: measurement of point i on the test scan.

n: total number of points measured in each analysis.

**Fig. 3:**
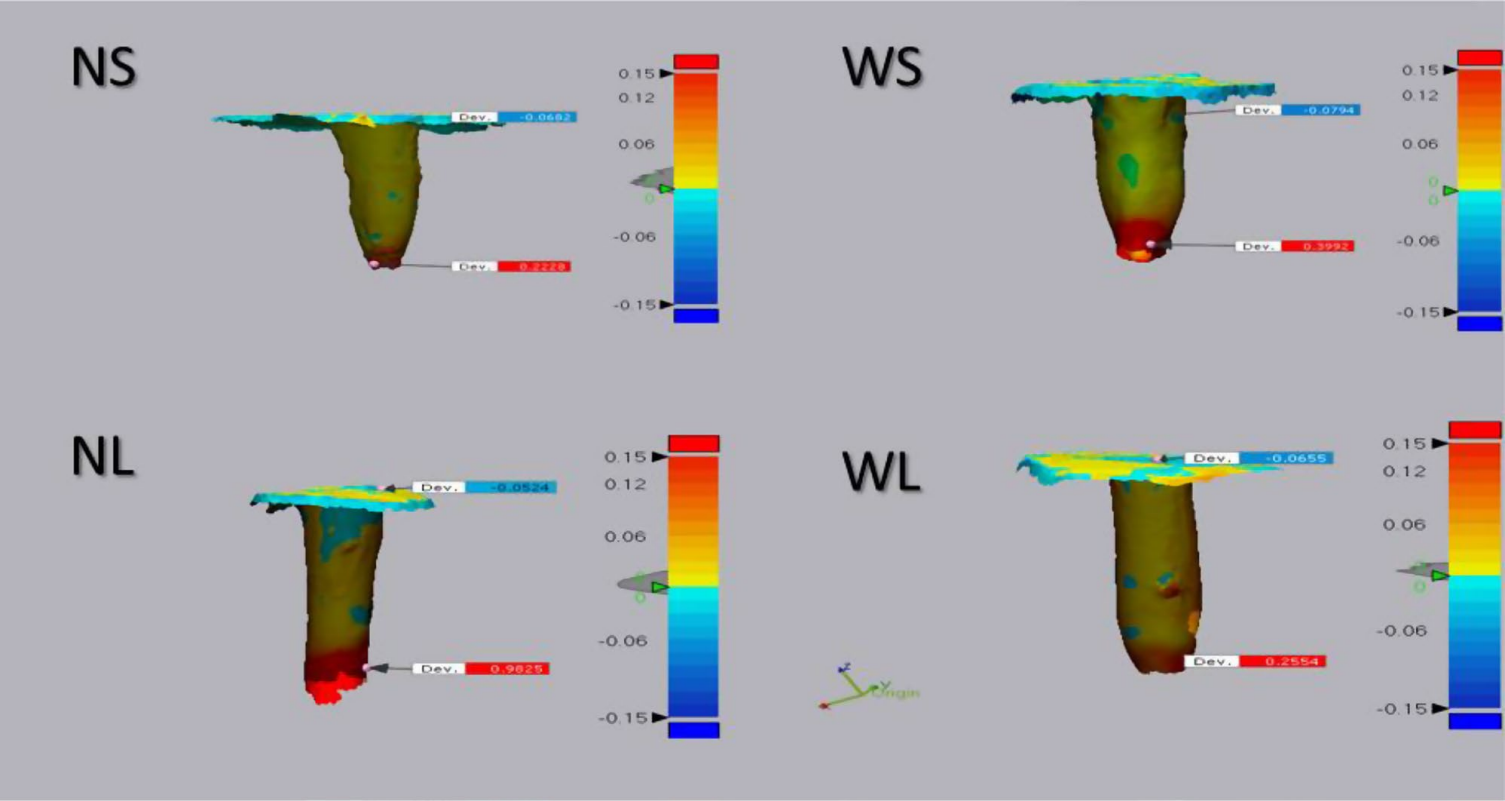
3D comparison of superimposed datasets showing deviations by color map

A color map was obtained with a deviation range of ± 0.15 mm, with tolerance set to zero. Green color indicated perfect matching, red color represented a relatively positive position in relation to the reference model, while blue color indicated a relatively negative position in relation to the reference model (Fig. 3).

### Depth discrepancy measurement

**Fig. 4:**
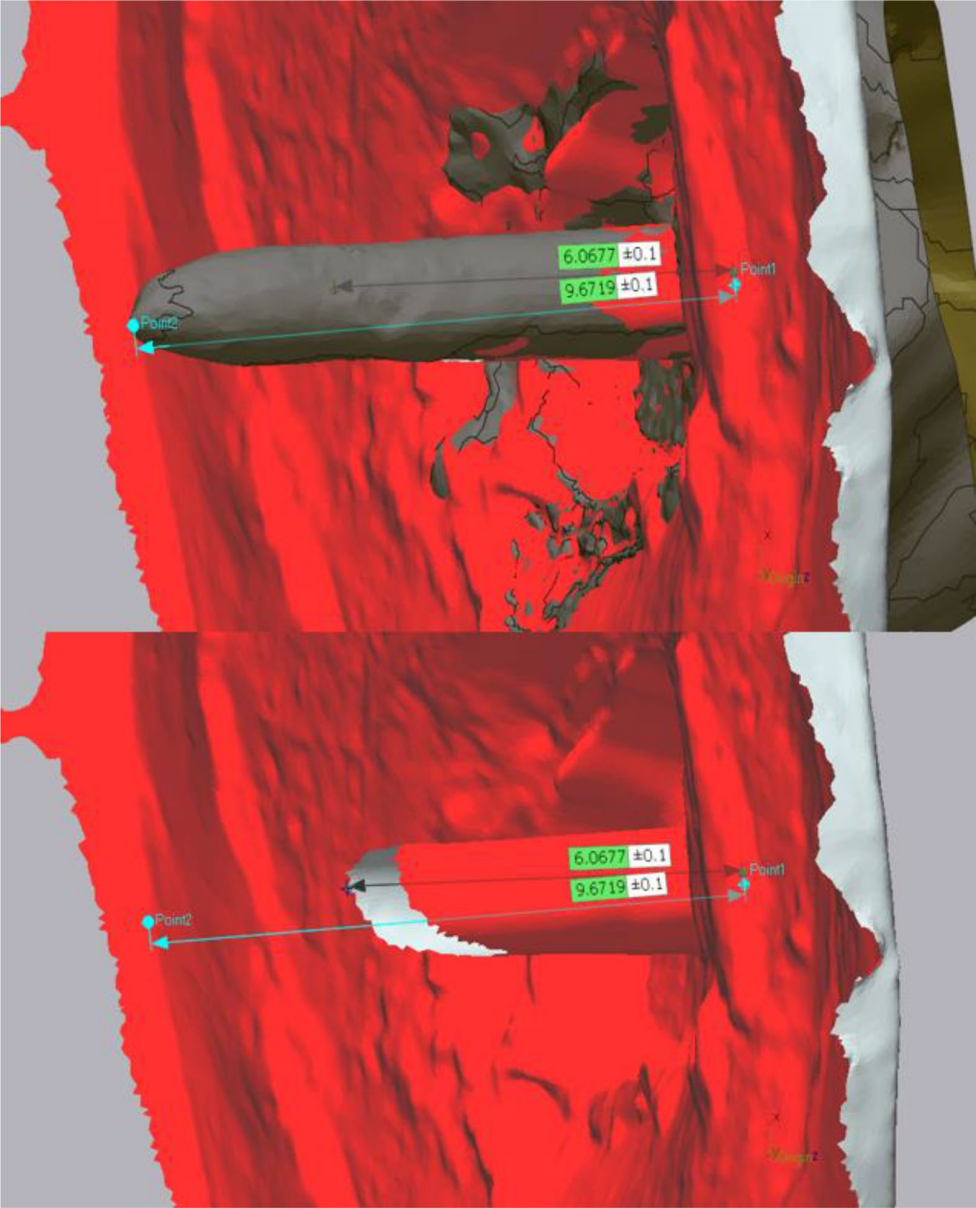
Depth discrepancy between Panda IOS and reference scanner datasets

After dataset alignment, three points on both the reference and imported datasets were assigned by two different examiners: one coronal point at the post space entry on the reference dataset and two apical points at the maximum extension of the post space on both the reference and imported datasets. Once the points were assigned, linear measurements of post space depths for both the reference and test datasets were obtained (Fig. 4). Post space depth discrepancy was calculated using the following equation:-.

$$\text{Depth Discrepancy}\,{\%}=\:\frac{Reference\:Depth-IOS\:Depth}{Reference\:Depth}\times\:100\%$$

### Statistical analysis

Descriptive statistics are presented as the mean and standard deviation for all subgroups. The normality of distribution was assessed using the Shapiro-Wilk test, and homogeneity of variances was evaluated with Levene’s test. The data followed a normal distribution and exhibited homogeneous variances. Statistical analysis of the depth discrepancy percentage was conducted using a one-sample t-test. Both trueness and depth discrepancy percentage data were analyzed for the effects of post space preparation width and depth using a two-way analysis of variance (ANOVA), followed by Tukey’s HSD for pairwise comparisons within the levels of the other factor. The correlations between post space width, depth, root mean square (RMS), and depth discrepancy percentage, as well as between depth discrepancy percentage and RMS, were evaluated using the Pearson correlation coefficient (PCC). A p-value of < 0.05 was considered statistically significant.

## Results

Root mean square (RMS) data are summarized in Table 2. The effect of preparation width on RMS was statistically insignificant for both short and long post space depths (*p* = 0.058 and *p* = 0.078, respectively). However, increasing preparation depth significantly reduced trueness (*p* < 0.05). In group (N), trueness was lower for NL (94.01 ± 10.79) than for NS (52.16 ± 4.08), while in group (W), WL (80.30 ± 7.55) exhibited reduced trueness compared to WS (42.80 ± 7.42). The interaction between post space preparation width and depth was statistically insignificant (*p* = 0.59), as shown in Table 3.

**Table 2:** Root mean square and depth discrepancy% of study groups

Preparation width	Preparation depth	RMS	Depth discrepancy%
Group (N)	Subgroup (NS)	52.16 ± 4.08	10.44 ± 3.26
	Subgroup (NL)	94.01 ± 10.79	33.16 ± 3.98
Group (W)	Subgroup (WS)	42.80 ± 7.42	4.32 ± 0.98
	Subgroup (WL)	80.30 ± 7.55	23.50 ± 2.25

N: Ø1.5 mm post space preparation; W: Ø1.7 mm post space preparation; NS: Ø1.5 mm, 6 mm post space preparation depth; NL: Ø1.5 mm, 10 mm post space preparation depth; WS: Ø1.7 mm, 6 mm post space preparation depth; WL: Ø1.7 mm, 10 mm post space preparation depth

Data presented as mean ± standard deviation (M ± SD)

**Table 3:** Two-way ANOVA results for the root mean square (RMS) values for trueness

RMS	Factors	Sum of Squares	df	Mean Square	F	P-value	F crit
	Width	665.28	1	665.28	8.30	0.01*	4.49
	Depth	7869.74	1	7869.74	98.16	<0.001*	4.49
	Width * Depth	23.74	1	23.74	0.30	0.59	4.49

df– degrees of freedom; * statistically significant (*p* < 0.05)

The correlations between preparation width and RMS for both short and long preparation depths were statistically insignificant (*p* = 0.058 and *p* = 0.078, respectively). However, preparation depth showed a significant, very strong positive correlation with RMS for both narrow and wide preparations (*r* = 0.935 and *r* = 0.959, respectively), suggesting that increasing preparation depth may increase RMS values.

The one-sample t-test indicated statistically significant depth discrepancy percentages across all subgroups. The depth discrepancy percentage was higher in the narrow preparation width group, with NS (10.44 ± 3.26) showing greater discrepancy than WS (4.32 ± 0.98), and NL (33.16 ± 3.98) exhibiting higher values than WL (23.50 ± 2.25) (*p* = 0.007 and *p* = 0.003, respectively). Preparation depth had a significant effect on depth discrepancy (*p* < 0.001), with NL (33.16 ± 3.98) displaying greater discrepancy than NS (10.44 ± 3.26), and WL (23.50 ± 2.25) showing higher values than WS (4.32 ± 0.98). The interaction between preparation width and depth was statistically insignificant (*p* = 0.23), as shown in Table 4.

**Table 4:** Two-way ANOVA results for depth discrepancy% values

Factors	Sum of squares	df	Mean square	F	*P*-value	F crit
Width	311.48	1	311.48	30.63	<0.001*	4.49
Depth	2194.25	1	2194.25	215.80	<0.001*	4.49
Width * Depth	15.62	1	15.62	1.54	0.23	4.49

df– degrees of freedom; * statistically significant (*p* < 0.05)

Preparation width demonstrated significant negative correlations with depth discrepancy for both short and long preparation depths (*r* = -0.785 and *r* = -0.831, respectively), indicating that increasing preparation width reduces depth discrepancy percentage. Conversely, preparation depth exhibited significant, very strong positive correlations with depth discrepancy for both narrow and wide preparation widths (*r* = 0.952 and *r* = 0.984, respectively), suggesting that greater preparation depth increases depth discrepancy.

**Fig. 5:**
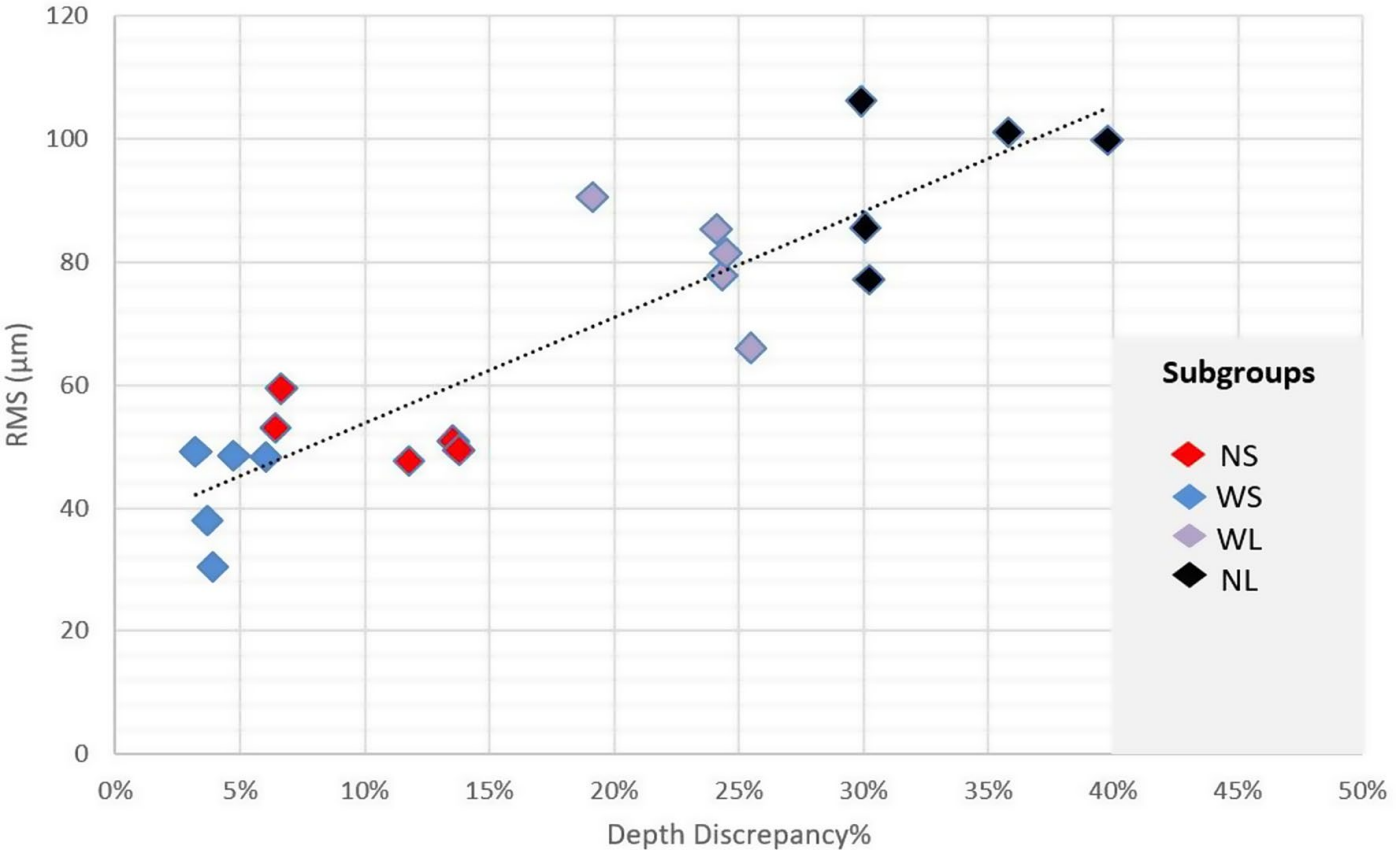
Scatter plot presenting the correlation between RMS and depth discrepancy%. RMS: Root mean square; NS: Ø1.5 mm, 6 mm post space preparation depth; NL: Ø1.5 mm, 10 mm post space preparation depth; WS: Ø1.7 mm, 6 mm post space preparation depth; WL: Ø1.7 mm, 10 mm post space preparation depth

Regarding the correlation between RMS and depth discrepancy of the Panda P2 IOS, a statistically significant, very strong positive correlation was observed (*r* = 0.898) (Fig. 5).

## Discussion

The null hypothesis was partially rejected, as preparation depth influenced both trueness and depth discrepancy percentage, while preparation width affected only depth discrepancy percentage without impacting trueness.

The introduction of resin cements has expanded restorative possibilities by offering superior mechanical and adhesive properties compared to conventional zinc phosphate cements. Additionally, advancements in restorative materials, such as dental ceramics and resins, alongside computer-aided design and manufacturing (CAD/CAM) technologies, have enabled the fabrication of durable restorations with reduced thickness and more complex geometries than traditional full-coverage crowns [49–51].

Tooth location within the dental arch significantly influences restoration success. Govare and Contrepois [52] reported favorable outcomes with endo-crowns in posterior teeth, where adequate support resists axial forces, with the adhesive properties of modern restorative materials and preservation of remaining dental tissues offering substantial advantages. In contrast, anterior restorations are exposed to significant masticatory and parafunctional forces, increasing the risk of fractures. Heintze [53] noted that the lack of mechanical retention in Class IV restorations presents adhesive challenges, leading to a failure rate nearly double that of Class III restorations. Comba et al. [54] found that resin composite restorations reinforced with intra-radicular retainers enhanced fracture resistance and minimized interfacial gap formation in incisors and canines, particularly when marginal ridges were compromised.

This study adopted the custom-made post-and-core approach to optimize fit. Pang et al. [55] reported that prefabricated posts with poor adaptation generate greater wedging forces and deflection within the post space, often leading to bonding failures and catastrophic fractures. The custom-made technique also preserves the existing ferrule width. Upadhyaya et al. [56] found that anatomical posts can be designed to maximize the ferrule effect without additional tooth structure removal, improving load distribution and reducing fracture risk. The anti-rotational effect is especially critical in restoring single-rooted teeth with extensive structural loss. Dangra and Gandhewar [57] emphasized that custom-made post-and-core systems provide superior resistance to rotational forces compared to prefabricated posts, which is crucial for single-rooted teeth prone to rotational stress during functional loading.

A uniform cement layer thickness is essential; Tsintsadze et al. [58] noted that a thin cement layer reduces polymerization stresses in custom-made restorations, a finding supported by Er [59]. Furthermore, bubble-free cementation in CAD-CAM custom-made posts contributed to void-free specimens in 80% of cases, as reported by Da Costa et al. [60], and bond strength can be enhanced with anatomical post-and-core systems [58, 61, 62].

Intraoral scanners have become a viable alternative to traditional vinyl poly-siloxane impressions for final impressions in clinical practice. Digital impressions offer advantages such as improved patient comfort especially for those with a pronounced gag reflex and the ability to selectively rescan unclear areas. Additionally, intraoral scanning reduces overall clinical treatment time by minimizing impression remakes and polymerization delays [63–65].

Intraoral scanners facilitate direct data acquisition from prepared abutments, reducing both procedural time and potential errors. Accurate digital scans are crucial for fabricating precise dental restorations. CAD/CAM systems have been used to mill fiber-reinforced composites and zirconia, enabling the creation of anatomical post-and-core restorations that fit elliptical post spaces with high accuracy [66].

In this study, post spaces were prepared with varying cervical widths and depths. Sequential water-cooled drilling was performed using a No. 1 Peeso reamer, gradually progressing from No. 2 to No. 5 for narrow post spaces and No. 6 for wider spaces. Farid et al. [67] emphasized that proper reamer selection preserves dentin while preparing post spaces. Preparation depths were standardized at 6 mm and 10 mm for the respective subgroups [68].

The Panda P2 intraoral scanner, utilizing continuous stereographic photography and a 45-degree scanning tip, was used. Sorrentino et al. [69] noted that this angulation enhances light reflection from vertical preparations, reducing software image adjustments and minimizing algorithmic errors.

Scanning was performed at room temperature under ceiling lighting below 1000 lx, per Maiti et al. [70], who highlighted the importance of controlled illumination for consistency. The scanning distance was set at 10 mm, following Rotar et al. [48], who found this distance optimal for intraoral scanner accuracy.

Conventional impressions were obtained using Hydrorise light-body polyvinyl siloxane (Zhermack, Italy), selected based on Re et al. [71], who reported its high ultimate strain at break (90.39 mm). This elasticity ensures structural integrity during retrieval from complex oral structures.

The impression technique in this study followed Elter et al. [28], utilizing light-body polyvinyl siloxane with a custom-sized wooden toothpick to maintain stability and prevent distortion.

Reference STL files were created by digitizing silicone impressions with the InEos X5 desktop scanner (Dentsply Sirona, Germany), per Emam et al. [45] and Elter et al. [28]. The InEos X5, chosen for its accuracy (trueness < 15 μm), exhibited the highest precision, with a trueness of 0.0 ± 1.9 μm according to Nulty [72]. Digital impressions were obtained first to prevent silicone residue from affecting post-space depth accuracy [45].

STL files were analysed using reverse engineering software. Each dataset was superimposed onto its reference using alignment algorithms. Geomagic Control X measured trueness and depth discrepancy percentage, following previous studies [28, 29, 45, 48, 68, 73–78].

Reference best-fit alignment minimized errors, focusing on the least deviated regions, excluding the post space. O’Toole et al. [79] found that this method significantly reduced measurement errors, yielding six times smaller translational errors and half the angular errors compared to conventional best-fit methods.

3D comparison analysis involved superimposing surfaces post-alignment, a method widely used in research [28, 29, 45, 48, 68, 73–78]. According to ISO 5725-1:2023 (paragraph 3.6), trueness reflects the closeness between test results and the reference value, indicating systematic errors. Bias, defined in paragraph 3.8, is the difference between the expected test result mean and the true reference value [47]. The RMS value is considered more reliable than the arithmetic mean, as it prevents cancellation of positive and negative deviations, avoiding misleadingly low deviation measures [80].

Depth discrepancy analysis followed Emam et al. [45], using STL file imports and the “2D length measurement” tool for post-space depth determination. Two independent examiners recorded the average values to minimize bias, as recommended by Pawar et al. [81]. Depth discrepancy was expressed as a percentage, following Pinto et al. [27], who compared post-space depth discrepancies between intraoral scanning and conventional impressions.

The effect of post-space preparation width on the trueness of the Panda P2 IOS was statistically insignificant, regardless of depth. This may be due to the limited additional scanning data or resolution gain from increased width. Although the small scanning tip (19.6 × 14.6 mm) improves accessibility, it may struggle to capture reflections at critical angles in wider preparations. Thus, any potential trueness improvement from additional data could be offset by increased image stitching errors (higher RMS). This aligns with An et al. [82], who found that smaller scanning tips reduced both trueness and precision, and Thanasrisuebwong et al. [83], who reported that larger scanning tips collected more data, improving trueness.

This study found that increasing post-space depth negatively affected trueness, supporting Hegazi et al. [84], who reported reduced trueness when increasing depth from 7 mm to 10 mm with Primescan AC IOS. Similarly, Almalki et al. [68] observed higher RMS in the apical third at 10 mm depth, and Elter et al. [28] found reduced trueness with deeper post spaces in mandibular canines using Primescan AC IOS. However, these results contrast with Emam et al. [45], who reported improved trueness and reduced RMS at greater depths for Primescan AC IOS, Medit i500 IOS, and CS3600 IOS.

The negative impact of increased depth on trueness may be due to limited light reaching the full depth, reducing reflections captured by the scanner and increasing RMS. Rotar et al. [48] noted that intraoral scanner light intensity decreases with greater scanning distance, reducing trueness. Additionally, as depth increases while diameter remains constant, incidence angles widen, lowering grazing angles and diminishing light capture. Londoño et al. [85] illustrated how extreme reflection angles hinder scanning accuracy. This grazing light phenomenon in the apical region may exaggerate surface texture and shadows, as reported by Sun [86], leading to missing data points and increased RMS.

Elter et al. [28] supported this, noting the greatest deviations in apical post-space regions. Similarly, Almalki et al. [68] found RMS at the apical third reached 133 μm at 10 mm depth. In contrast, Emam et al. [45] reported improved trueness with increasing depth, potentially due to different preparation designs. Their study used a tapered Olipost drill (Ø1.6 mm, Olident, Poland), which may have widened post spaces with increasing depth, influencing trueness.

Despite variations, RMS values for all scanned post spaces remained below 100 μm, within the clinically acceptable range (50–100 μm) for adequate cement gaps during cementation or bonding [68, 87].

Depth discrepancy percentage findings align with Pinto et al. [27], who noted that post-space depth acquisition depends on scanner hardware, software, and preparation width. For the Panda P2 IOS, increased width improved depth acquisition regardless of preparation depth, likely due to enhanced light entry, optimized incidence angles, and reduced light entrapment, as supported by Fu and Shi [88] and Gerasimov et al. [89].

One might question why preparation width significantly affected depth acquisition but not RMS. This discrepancy can be explained by the different assessment methods: trueness is measured by deviations from a reference, while depth discrepancy percentage depends on the linear post-scan extent, independent of deviations or resolution.

The effect of depth on depth discrepancy percentage partially agrees with Emam et al. [45], who found a significant difference between post-scan length and actual depth in CS3600 IOS, with greater discrepancies at 10 mm compared to 8 mm. The disagreement may stem from tapered preparation designs, where increased depth also means increased width, influencing results.

The relationship between depth and depth discrepancy percentage can be attributed to the same factors discussed earlier. Rotar et al. [48] found that deeper preparations receive less light, reducing reflection capture and leading to missing data points. Sun [86] described how light grazing in the apical region accentuates surface textures, creating exaggerated shadows that obstruct calculations and form missing data areas.

The clinical significance of depth discrepancy percentage aligns with Perucelli et al. [90], who emphasized that the apical segment of post-and-core restorations should maintain direct contact with residual gutta-percha to prevent micro-leakage and bacterial infiltration. An apical gap exceeding 2 mm is associated with clinical complications, a threshold also supported by Hendi et al. [13] and Jafarian et al. [91]. Based on this limit, the clinically acceptable depth discrepancy percentage is 33.3% for a 6 mm post space and 20% for a 10 mm post space. All study subgroups fell within these limits, except NL and WL, which exceeded the 20% threshold for 10 mm post spaces [13, 90, 91].

The strong positive correlation between depth and depth discrepancy percentage may be attributed to shared anatomical factors affecting light exposure and reflection, ultimately influencing light capture and processing.

This study has limitations, as digital scans were performed in a controlled, non-clinical setting, which may not fully replicate intraoral conditions such as saliva contamination, patient movement, or light reflections from intraoral structures. Another limitation is the use of Peeso reamers for post-space preparation without a finishing step, potentially introducing surface irregularities. These irregularities may have influenced light interaction, compromising digital impression trueness. Future studies should explore the effect of post-space wall treatment and finishing in reducing these irregularities, which may enhance both trueness and reproducibility in intraoral scanner-based post-endodontic restorations.

Advancements in intraoral scanning technology may further improve post-space scanning accuracy. Enhanced scanner resolution and AI-driven algorithms could minimize distortions and data loss, leading to more precise digital impressions. Additionally, varied scanner tip dimensions could improve accessibility and provide a larger capture window, enhancing scan accuracy in different clinical scenarios. Long-term clinical studies comparing intraoral scanning with conventional impression techniques are needed to validate digital workflows and assess their impact on prosthetic success.

## Conclusions

Within the limitation of this study, it could be concluded that:

1. Post space width had no significant effect on trueness at any depth but improved depth acquisition by reducing depth discrepancies. On the other hand increasing preparation depth significantly reduced trueness and increased depth discrepancy percentage.
2. There was no significant interaction between post space width and depth.
3. The Panda P2 IOS exhibited clinically acceptable trueness across all subgroups; however, at a preparation depth of 10 mm, the apical gap (depth discrepancy percentage) exceeded the clinically acceptable limit of 2 mm.
4. A strong positive correlation was found between root mean square and depth discrepancy percentage.

## Data Availability

The datasets generated and analysed during the current study are available from the corresponding author on reasonable request.

## Abbreviations

IOS: Intraoral scanner
CAD-CAM: Computer aided design and computer aided manufacturing
STL: Standard tessellation language
RMS: Root mean square
PCC: Pearson’s correlation coefficient
